# Microbial melatonin metabolism in the human intestine as a therapeutic target for dysbiosis and rhythm disorders

**DOI:** 10.1038/s41522-024-00605-6

**Published:** 2024-11-27

**Authors:** Petra Zimmermann, Salome Kurth, Benoit Pugin, Nicholas A. Bokulich

**Affiliations:** 1 Department of Community Health, Faculty of Science and Medicine, University of Fribourg, Fribourg, Switzerland; 2 Department of Paediatrics, Fribourg Hospital, Fribourg, Switzerland; 3 Infectious Diseases Research Group, Murdoch Children’s Research Institute, Parkville, VIC Australia; 4 Department of Paediatrics, The University of Melbourne, Parkville, VIC Australia; 5 Department of Psychology, University of Fribourg, Fribourg, Switzerland; 6 Laboratory of Food Systems Biotechnology, Department of Health Sciences and Technology, ETH Zurich, Zurich, Switzerland

**Keywords:** Microbiome, Clinical microbiology

## Abstract

Melatonin (MT) (N-acetyl-5-methoxytryptamine) is an indoleamine recognized primarily for its crucial role in regulating sleep through circadian rhythm modulation in humans and animals. Beyond its association with the pineal gland, it is synthesized in various tissues, functioning as a hormone, tissue factor, autocoid, paracoid, and antioxidant, impacting multiple organ systems, including the gut-brain axis. However, the mechanisms of extra-pineal MT production and its role in microbiota-host interactions remain less understood. This review provides a comprehensive overview of MT, including its production, actions sites, metabolic pathways, and implications for human health. The gastrointestinal tract is highlighted as an additional source of MT, with an examination of its effects on the intestinal microbiota. This review explores whether the microbiota contributes to MT in the intestine, its relationship to food intake, and the implications for human health. Due to its impacts on the intestinal microbiota, MT may be a valuable therapeutic agent for various dysbiosis-associated conditions. Moreover, due to its influence on intestinal MT levels, the microbiota may be a possible therapeutic target for treating health disorders related to circadian rhythm dysregulation.

## Introduction

Melatonin (MT) (N-acetyl-5-methoxytryptamine) is an indoleamine that functions as a hormone, tissue factor, autocoid, paracoid, and antioxidant^1^. In mammals, including humans, MT is predominantly synthesized in the pineal gland and regulates circadian rhythms^2^. However, MT can also be synthesized by enterochromaffin cells and bacteria in the intestine. Factors that stimulate MT production in the intestine include the intestinal microbiota and their metabolites (such as short-chain fatty acids), food intake, immune responses, and mitochondrial function^3^. As MT has been shown to have positive effects on various diseases, including inflammatory bowel disease, obesity, and neuropsychiatric disorders, there may be a significant link between intestinal microbiota and MT. These insights highlight the complex interplay between diet, microbiota, and physiological processes in regulating intestinal MT levels, which could have important implications for treating gastrointestinal and systemic diseases.

This review summarizes how MT affects the intestinal microbiota, explores the microbiota’s contribution to intestinal MT levels, and discusses the relevance of intestinal MT production for human health.

### Evolution

MT is one of the earliest compounds to have emerged in living organisms for coordinating essential functions and is phylogenetically one of the most conserved molecules in biology^4^. In unicellular organisms, MT primarily serves as an antioxidant^5,6^. Its biosynthetic machinery in eukaryotes likely originated through endosymbiosis of bacteria^7^, possibly *Rhodospirillum rubrum*, which is considered a potential precursor of mitochondria^7^. *R. rubrum* is one of the oldest known bacteria and also the first bacteria discovered to produce MT^8^. As multicellular organisms evolved, MT became a signaling molecule that translates environmental light/dark cycles into endocrine signals^9^. Its receptor-mediated functions were acquired during evolution^10^. MT is found not only in humans but also in many other animals^11^ and plants^12,13^.

### History

MT was first isolated from the bovine pineal gland less than 70 years ago, in 1958, by dermatologist Aaron Lerner^14^, who named it for its ability to lighten frog skin by affecting melanophores and its chemical relation to serotonin^15,16^. Lerner later was also the first to isolate MT from humans^17^. The discovery that MT levels vary between day and night was made in rats in 1964^18^. It took another decade until it was discovered in mice that exposure to light decreases MT production^19^.

## MT production and physiological pathways in humans

In mammals MT is predominantly synthesized in the pineal gland during dark periods^2^. Light information is transmitted from the retina to the suprachiasmatic nucleus (SCN) via the retinohypothalamic tract. Specialized photoreceptors in the retina, such as melanopsin-containing ganglion cells, detect light and relay signals to the SCN^20,21^. The SCN then sends regulates MT production by sending signals to the pineal gland, which does not perceive light directly. Additionally, the SCN receives hormonal signals from the raphe nuclei (serotonin, which sets the circadian phase and body vigilance) and the pineal gland (MT, which facilitates sleep)^22^. Accordingly, the nocturnal MT production varies seasonally, with longer peaks during winter nights and shorter peaks during summer^23^. MT’s primary role in humans is regulating circadian rhythms, body temperature, the sleep-wake cycle, cortisol secretion, and blood pressure^24^. However, it also affects various other organ systems, including the cardiovascular and reproductive systems^25–28^, body mass index^29^, and bone growth^30,31^.

Beyond its role as a circadian pacemaker, MT serves as a potent antioxidant and immunomodulator by influencing key regulators such as nuclear factor-kappa B (NF-kB), signal transducer and activator of transcription, NOD-like receptor family pyrin domain containing 3 inflammasome, mitogen-activated protein kinase, and toll-like receptors^32–36^. It restores glutathione homeostasis and exhibits antiapoptotic and oncostatic effects^37–39^. MT also influences electrolyte transport across membranes^40^ and has chelating properties, reducing the cytoplasmic availability of metals^41^. Substantial amounts of MT are found in tissues and organs frequently exposed to environmental stress, such as the intestine and skin, or those with high oxygen consumption, like the brain^42^. Additionally, MT production may be upregulated by low-intensity stressors such as dietary restriction or exercise^43,44^.

In the human epithalamus, pinealocytes take up tryptophan from the blood and convert it to serotonin through hydroxylation and decarboxylation. Serotonin is then acetylated into N-acetyl-serotonin by the rate-limiting enzyme arylalkylamine N-acetyltransferase (AA-NAT) (formerly referred to as serotonin N-acetyl transferase), whose activity is upregulated during darkness. Finally, N-acetyl-serotonin is methylated into MT by the enzyme N-acetylserotonin O-methyltransferase (ASMT)^45^. MT is an amphiphilic molecule, possessing both lipophilic and hydrophilic properties^46^. In blood, ~70% of MT is bound to albumin^47^. MT is primarily metabolized in the liver by P450 monooxygenases (CYP1A2) and then conjugated with sulfate or glucuronic acid to become more water-soluble, facilitating excretion into urine, mostly as 6-sulfatoxyMT^48^ or N1-acetyl-N2-formyl-5-methoxykynuramine^42^.

Although MT can be produced in the cytoplasm, it is primarily synthesized within mitochondria^7,49^. It performs several critical functions at the mitochondrial level, such as scavenging reactive oxygen species^50^, helping maintain mitochondrial membrane potential, supports adenosine triphosphate synthesis, and enhances the efficiency of the electron transport chain, regulates mitochondrial dynamics by promoting fusion and reducing fission, which is crucial for maintaining mitochondrial function and cell survival^50–52^. It also promotes mitophagy, aiding in the removal of damaged mitochondria and maintaining mitochondrial homeostasis^50^.

On the systemic level, MT exerts its effects primarily through interaction with G protein-coupled receptors (GPCRs): MT1 and MT2 receptors. MT1 receptors are widely distributed in the central nervous system, particularly in the SCN of the hypothalamus, the central circadian pacemaker^53^. MT1 receptors are also found in the retina^54^, pituitary gland^55^, and other peripheral tissues, including the cardiovascular system^28^. MT1 receptors regulate circadian rhythms, which affects sleep and reproductive physiology ^53^. MT2 receptors, also present in the SCN, are more widely distributed in peripheral tissues than MT1 receptors^53^. They are present in the retina, various brain areas, and peripheral tissues such as the kidneys, liver, and cardiovascular system^28^. MT2 receptors modulate phase shifting of circadian rhythms (e.g., adjusting the biological timing during travel across time zones), retinal functions, glucose metabolism, and immune responses^56^. Additionally, there is a MT3, which is not a traditional GPCR but a cytosolic enzyme called quinone reductase 2^57^. It is found in various tissues, including the liver, kidney, brain and intestine^56,58^. Its role in MT signaling is less clear but may involve detoxification and protection against oxidative stress.

## MT production by the intestine and its relation to food intake and circadian rhythm

In animals, MT synthesis occurs in various body parts beyond the pineal gland, including the retina^59^, Harderian gland^60^, skin^61^, bone marrow^62^, macrophages^63^ and the intestine^64–66^. In contrast, evidence for extrapineal MT production in humans is limited^54,62,67^. Some animals studies suggest that after pinealectomy, circadian rhythm of MT levels in blood perist^68–72^, while others report its abscence^73^. Due to missing habituation to the light-dark cycle and its effects on MT dynamics some but not all visually impaired humans encounter circadian desynchrony and sleep disturbances^74^. The concept of intestinal MT production emerged with the identification of MT-producing enzymes in the intestine^75,76^. Debate continues regarding whether enterochromaffin cells in the intestine produce MT^67,77^. In animals, MT has been detected from the esophagus to the rectum with the highest levels in the small intestine^78,79^. Levels of MT in the intestine have been reported to be 400 times higher than in the pineal gland^67,80^ and 10–100 times higher than in the blood^70,78–80^, although this has recently been questioned due to the uncertainties in methodological issues with measuring MT^67^. Similar findings of over 10 times higher MT levels in the intestine compared to circulation have been reported in humans^81^. Furthermore, MT levels are higher in portal blood than in peripheral venous blood supporting the idea of MT production in the gastrointestinal tract^82^. While intestinally produced MT can increase serum levels of MT^3^, it remains unclear how much enters into the cerebrospinal fluid. Therefore, while intestinal MT can have systemic effects, it does not replicate the function of pineal melatonin in regulating the circadian rhythm.

In animals, fluctuations in intestinal MT production have been linked to food intake^83–85^. Specifically, tryptophan administration can increase intestinal production and levels of MT^80,86,87^. Interestingly, oral administration of L-tryptophan leads to a rapid increase in circulating MT, which is abolished by partial ligature of the portal vein, indicating MT synthesis in the intestine^80,88^. In humans, diets rich in tryptophan (e.g., white meat, dairy) and MT (e.g., eggs, fish, nuts, some mushrooms, cereals, germinated legumes, wine) also increase MT levels^89–92^. It has been suggested that the increased MT levels found after tryptophan administration may be due to extrapineal production, though evidence remains controversial^93^. Interestingly, food deprivation has been shown to increase levels of tryptophan, serotonin, and MT in the intestine of mice^94^. In humans, however, fasting has been observed to decrease nocturnal circulating MT levels^44^. These studies highlights that temporal dynamics of MT levels (both intestinal and peripheral) in relation to food intake and fasting still need further elucidation.

Recent evidence shows that the intestinal microbiota and its metabolites exhibit diurnal rhythmicity, primarily responding to feeding/fasting cycles^95–98^. There is a bidirectional relationship where the host’s circadian rhythms influence the gut microbiome, and the microbiome, in turn, affects the host’s circadian clock^97,99,100^. Microbially derived mediators, such as short-chain fatty acids and bile acids, have been identified as potential signals between the gut microbiome and host circadian clocks^101^, but it is likely that MT contributes to this.

## MT production and regulation by bacteria and fungi

Intestinal microbiota play crucial roles in regulating serotonin biosynthesis and tryptophan metabolism in the intestine^102–104^. The availability of intestinal tryptophan can be altered by changes in microbiota composition, as seen in germ-free mice^105^. Microbiota-derived metabolites, such as bile acids and short-chain fatty acids, stimulate enterochromaffin cells to release serotonin, potentially enhancing MT production^106,107^. In mice, dysbiosis (disruption in the normal composition of the microbial communities) and germ-free conditions have been shown to directly influence local and systemic MT levels^98,108,109^.

Enterohepatic recirculation of MT is one mechanism by which intestinal microbiota may regulate circulating levels of MT. As previously mentioned, circulating MT is metabolized in the liver via decarboxylation and conjugation (with glucuronic acid or more typically sulfate in humans), allowing excretion in urine or into the intestine via biliary excretion^110–113^. These compounds could then be deconjugated by microbial enzymes in the intestine, leading to reabsorption and return to circulation^114^, as observed with other glucuronate- and sulfate-conjugated compounds that undergo enterohepatic recirculation^114,115^.

A few bacteria and fungi also have been shown to synthesize MT (Table 1). The first discovery of bacterial MT production was made in 1995 with *R. rubrum*, an organism estimated to be 2 to 3.5 billion years old. It was shown that this bacterium produces significantly more MT during extended periods of darkness compared with light periods^8^. Similarly, *Porphyrobacter* (formerly *Erythrobacter*) *longus* has been shown to produce more MT during darkness^116^. It has been hypothesized that in bacteria, this variation is passive rather than due to reduced synthesis, possibly linked to depletion from utilization. During daylight hours, photosynthetic organisms may metabolize more MT due to its interaction with reactive oxygen species that are generated in high quantities during photosynthesis. Another example of a MT-producing cyanobacterium is *Arthrospira platensis* (better known as “*Spirulina*”)^117^. Two bacteria colonizing grape plants (*Bacillus amyloliquefaciens* and *Pseudomonas fluorescens*) have also been found to produce MT. MT was associated with enhanced plant growth, reduction in reactive oxygen species and mitigation of cell damage^118^. MT production was upregulated when the plants were exposed to salt or drought stress (Table 1).

**Table 1:** Bacteria and fungi that have been identified to produce MT

Bacteria	Important findings
*Bacillus amyloliquefaciens*	Colonization of grapevine plantlets by B*. amyloliquefaciens*: • increases plant growth^214^ • mitigates adverse effects of salt and drought stress by reducing production^214^ of malondialdehyde and reactive oxygen species in plant roots^214^ • when grapevine plantlets are exposed to salt or drought stress there is an upregulation of MT synthesis and its intermediates^214^
*Rhodospirillum rubrum*	• higher production in bacteria grown in the dark^8^
*Pseudomonas fluorescens*	Colonization of red globe grape roots by *P. fluorescens:* • increased plant growth^118^ • increased levels of 5-hydroxytryptophan, N-acetylserotonin, and MT^118^ while decreasing levels of tryptamine and serotonin under salt stress, correlating with reduced reactive oxygen species and mitigated cell damage^118^
*Porphyrobacter* (formerly *Erythrobacter*) *longus*	• higher production in bacteria grown in the dark^116^
*Arthrospira (*formerly *Spirulina) platensis*	• serotonin N-acetyltransferase and hydroxyindole-O-methyltransferase found in *S. platensis*^117^

Fungi	Important findings
*Metschnikowia pulcherrima*	• produced during fermentation^119^
*Saccharomyces cerevisiae*	• produced during fermentation^119^ • decreased MT production in a salt medium^120^ • adding 5-methoxytryptamine to cultures increases MT production^120^ • higher MT production in medium with low levels of reducing sugars^121^ • MT synthesis depended on the yeast growth phase, tryptophan level, reducing sugar levels and growth medium^121^
*Saccharomyces uvarum*	• MT synthesis depended on the yeast growth phase, tryptophan level, reducing sugar levels and growth medium^119^
*Tolypocladium guangdongense*	• increased MT production in response to stress imposed through Congo red, cold, and heat^215^ • decreased levels of MT intermediates in response to Congo red^215^ • key MT synthesis gens upregulated early under stress through Congo red but downregulated later^215^
*Torulaspora delbrueckii*	• produced during fermentation^119^

Several fungi have been found to produce MT during fermentation processes (*Metschnikowia pulcherrima, Saccharomyces cerevisiae, Saccharomyces uvarum*, and *Torulaspora delbrueckii*) (Table 1)^119–121^. The synthesis of MT in these fungi was influenced by growth phase, tryptophan levels, reducing sugar levels, and the composition of the growth medium^120,121^. *S. cerevisiae*, commonly known as baker’s yeast, is occasionally found in the human intestinal microbiota^122–124^. *M. pulcherrima* and *T. delbrueckii* are less likely to be found in the human intestinal microbiota, as they are typically associated with other environments such as fruits, soil, or fermenting plants^125^.

Little is known about the enzymes involved in MT biosynthesis in microorganisms. However, some have been identified in bacteria, such as AA-NATin the cyanobacterium *Synechocystis* sp. PCC 6803^126^ and several *Staphylococcus* spp^127^. These enzymes can also acetylate tryptamine, a tryptophan catabolite and serotonin precursor produced by intestinal microbes^104^. In *S. cerevisiae*, an AA-NAT has been identified^128^. Interestingly, the biochemical characterization of certain AA-NAT enzymes revealed higher substrate affinity with 5-methoxytryptamine (5-MT) than serotonin^126,128^, suggesting that an alternative pathway, where serotonin is first O-methylated to 5-MT and then N-acetylated to MT, may be predominant in microorganisms^129^.

## Influence of MT on bacteria and fungi

It has been shown that MT enhanced the swarming activity of the intestinal bacteria *Klebsiella aerogenes* (formerly *Enterobacter*), exhibiting a rhythmic pattern with a circadian period of ~24 h^130^. Recently, a study found that MT increases the size of *K. aerogenes* colonies and synchronizes its circadian clock in a dose-dependent manner^131^. Exposure to MT induced the expression of 81 transcripts during exponential growth and 30 transcripts during early stationary phase. These transcripts predominantly included genes involved in biofilm formation, fimbria biogenesis, transcriptional regulation, carbohydrate transport and metabolism, phosphotransferase systems, stress response, and metal ion binding and transport. Notably, the differential expression of biofilm and fimbria-related genes suggests that MT might enhance *K. aerogenes’* capacity for host colonization (Table 2).

**Table 2:** Summary of findings of studies investigating the effect of MT on bacteria and fungi

Bacteria	Important findings
*Acinetobacter baumannii*	• inhibited growth^132^
*Klebsiella* (formerly *Enterobacter) aerogenes*	• MT causes an increase in the swarming activity of *K. aerogenes* which occurs rhythmically with a period of ~24 h^130^ • possible coordination between circadian clocks of host (mammals) and microbiota (such as *K. aerogenes*) in intestine^130^ • MT increases the size of macrocolonies (clusters of bacterial cells) on semisolid agar and synchronizes the circadian clock of *K. aerogenes* in a level-dependent manner^131^ • 81 transcripts showed differential expression during exponential growth and 30 transcripts showed differential expression during early stationary phase in response to MT^131^ • MT-sensitive genes are involved in various biological processes such as biofilm formation, fimbria biogenesis, transcriptional regulation, carbohydrate transport and metabolism, phosphotransferase systems, stress response, and metal ion binding and transport^131^ • MT might enhance the ability of *K. aerogenes* to colonize its host potentially influencing its ecological role in the intestine^131^
*Mycobacterium tuberculosis*	• inhibited growth^133^
*Mycobacterium bovis*	• inhibited growth^133^
*Pseudomonas aeruginosa*	• inhibited growth^132^
*Staphylococcus aureus*	• inhibited growth^132^
*Xanthomonas oryzae*	• inhibited growth, motility, and biofilm formation^134,135^ • reduced mRNA expression of genes involved in cell division, toxin production, carbohydrate and amino acid metabolism^134,135^

Fungi	Important findings
*Saccharomyces cerevisiae*	• MT supplementation attenuates the negative effects of ethanol on *S. cerevisiae* (reduced growth, increased mortality rates, higher levels of reactive oxygen species, lipid peroxidation)^138^ • protective effects of MT varies depending on strain, level of MT, and growth phase^138^

**Fig. 1:**
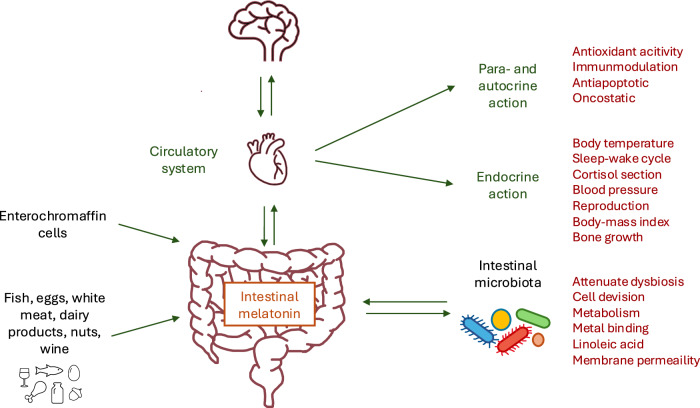
Intestinal melatonin and its effects.

In contrast to the increased colony sizes of *K. aerogenes*^130^, MT exposure inhibits growth of *Acinetobacter baumannii*^132^*, Mycobacterium tuberculosis*^133^*, Mycobacterium bovis*^133^ *Pseudomonas aeruginosa*^132^ *Staphylococcus aureus*^132^, and *Xanthomonas oryzae*^134,135^. In *X. oryzae*, MT has also been shown to lead to reduced mRNA expression of genes associated with cell division, toxin production, as well as carbohydrate and amino acid metabolism^134,135^. MT can inhibit bacterial growth by regulating gene expression associated with cell division^134,135^ and metabolism^135^, reducing cytoplasmic availability of metal ions^41^, restricting linoleic acid absorption (used for cell proliferation)^136^, and enhancing bacterial outer membrane permeability (Fig. 1)^137^.

A further example that MT is produced in response to environmental stress can be taken from fungi. During alcoholic fermentation, *Saccharomyces cerevisiae* faces various stresses, with ethanol being particularly significant. MT supplementation attenuates the negative effects of ethanol on *S. cerevisiae* (reduced growth, increased mortality rates, higher levels of reactive oxygen species, and lipid peroxidation)^138^. The protective effect varies depending on the strain, MT level, and growth phase.

## Influence of MT on the composition of the intestinal microbiota

In total, 14 studies investigated the effect of MT on the composition of the intestinal microbiota in 663 mice (mean 44, range 8–96 per study) (Table 3)^108,109,139–149^. This included mice exposed to toxins (aflatoxin B *n* = 6^139^, ochratoxin A *n* = 60^150^) or to stress (sleep deprivation *n* = 87^108,109^, water *n* = 12^144^, water and sleep deprivation *n* = 12^144^, restraint *n* = 24^142^, jet-lag *n* = 12^151^), with chemically-induced colitis (oxazolone *n* = 8^141^, dextran-sodium sulfate *n* = 100^140,143,147^), with spinal cord injury (*n* = 44)^145^, receiving a high-fat diet (*n* = 30)^146,149^, receiving antibiotics (*n* = 35)^148,150^, only receiving MT (*n* = 64)^142,143,145,148,151^, as well as germ-free (*n* = 20)^148^ and control mice (*n* = 139)^108,109,139,140,142–146,148–151^. In total, 298 (45%) mice received MT, which was injected intraperitoneally (*n* = 172)^108,109,142–145^, given orally (*n* = 117)^139,140,146–151^ or injected into the colon (*n* = 9)^141^. The doses of MT can be found in Table 1.

**Table 3:** Summary of findings of studies investigating the effect of MT on the composition and function of the intestinal microbiota

Author Study population Country Publication year	Characteristics and housing conditions of animals Number of animals and interventions	Sample type DNA extraction kit Microbiota analysis technique Sequenced region and primers Sequencing platform Sequencing length, depth Database for taxonomic identification Database for metabolic pathways	Effect on the microbiota and metabolic pathways	Other findings
Liu et al.^139^ Mice exposed to aflatoxin B1 China 2022	6w-old male C57BL/6 mice, standard diet Light conditions nr 2 alfatoxin B1 (0.75 ng/kg/d) orally for 2w 2 alfatoxin B1 (0.75 ng/kg/d) and MT (20 mg/kg/d) orally for 2w 2 alfatoxin B1 (0.75 ng/kg/d), MT (20 mg/kg/d) and glycine-betaine-muricholic acid (10 mg/kg/d) orally for 2w 2 controls	Colon content DNeasy PowerSoil Kit (*Qiagen*) 16S rRNA gene sequencing V3, V4, nr nr nr, nr nr N/A	**Alfatoxin B1-exposed mice receiving MT (n** = **2) compared with alfatoxin B1-exposed mice without MT (n** = **2)** • Higher richness and diversity (number of OTUs, ACE, Chao, Shannon, and Simpson indices) • Lower relative abundance of Bacillota, and higher relative abundance of Bacteroidetes • Lower relative abundances of *Clostridiales* and *Lactobacillales* • Lower relative abundances of *Desulfovibrio, Clostridium*_XIVa, and *Lactobacillus*	**Alfatoxin B1-exposed mice receiving MT (n** = **2) compared with alfatoxin B1-exposed mice without MT (n** = **2)** • Higher expression of intestinal tight junction proteins (claudin-1, occluding, zonula occludens-1), and lower intestinal permeability in ileum • Lower levels of lipopolysaccharides in serum and liver (not in antibiotic-treated mice) • Higher levels of farnesoid X receptor protein and lower levels TLR4/NF-κB signalling pathway-related mRNA, as well as lower expression of TLR4, MyD88, p-p65, and p-IκB in ileum (not in mice treated with glycine-betaine-muricholic acid)
Jing et al.^140^ Mice with DSS-induced colitis China 2022	6 to 8w-old male C57BL/6 mice, standard diet Light conditions nr 8 3%-DSS in drinking water for 7 d 8 3%-DSS in drinking water for 7 d followed by 5-aminosalicylic acid (50 mg/kg/d) 8 3%-DSS in drinking water for 7 d followed by hyaluronic acid orally for 7 d 8 3%-DSS in drinking water for 7 d followed by MT orally for 7 d 8 3%-DSS in drinking water for 7 d followed by hyaluronic acid and MT orally for 7 d 8 controls	Stool Tianamp stool DNA kit (*Tiangen*) 16S rRNA gene sequencing V3, V4, 338 F/806 R Nr nr, nr Greengenes 13_8 N/A	**Mice with colitis receiving MT (n** = **16) ± hyaluronic acid compared to mice with colitis without MT (n** = **8)** • Higher richness and diversity (number of OTUs, Shannon index) • Higher relative abundance of Bacillota • Higher relative abundance of *Lactobacillus*, lower relative abundance of *Bacteroides, Blautia*, and *Streptococcus*	**Mice with colitis receiving MT (n** = **16) ± hyaluronic acid compared to mice with colitis without MT (n** = **8)** • Improvement of colitis symptoms, decreased colon weight/length ratio, decreased spleen size, alleviation of damaged intestinal barrier, decreased colon inflammation (more uniform crypt structure and decreased inflammatory cell infiltration) • Higher expression of intestinal tight junction proteins in colon (occluding, zonula occludens-1),
Zhao et al.^141^ Mice with oxazolone-induced colitis China 2022	6 to 8w-old male C57BL/6 mice, nr Light conditions nr 9 3% oxazolone (4.5 uL/g) injected into colon 9 MT (50 mg/kg/d) orally for 7 d before 3% oxazolone (4.5 uL/g) injected into colon	Stool EZNA soil kit (*Omega Bio-Tek)* 16S rRNA gene sequencing V3, V4, 338 F, 806 R MiSeq 2500 (*Illumina*) nr, nr nr N/A	**Mice with colitis receiving MT (n** = **6) compared with mice with colitis without MT (n** = **6)** • Lower richness and diversity (ACE, Chao, and Shannon indices) • Higher relative abundances of Verrucomicrobiota and Actinomycetota • Lower relative abundance of *Lachnospiraceae* and *Peptococcaceae* • Higher relative abundances of *Bifidobacterium and* lower relative abundances *of Desulfovibrio*	**Mice with colitis receiving MT (n** = **9) compared with mice with colitis without MT (n** = **9)** • Higher body weight, less colon shortening, and lower histological pathology score in colon • Lower levels of neutrophils, TNF-α, IL-1ß, IL-5, and IL-13 in colon • Higher expression of tight junction proteins (occludin, zonula occludens-1) in colon
Lin et al.^142^ Mice exposed to restraint stress China 2021	8w-old male IRC mice, standard diet 14 h light/10 h dark cycles 12 restraint for 3 d 12 restraint and MT (20 mg/kg/d) intraperitoneally for 3 d 12 MT (20 mg/kg/d) intraperitoneally for 3 d 12 controls	Colon content DNeasy PowerSoil Kit (*Qiagen*) 16S rRNA gene sequencing V3, V4, 338 F, 806 R HiSeq 2500 (*Illumina*) 2×250, mean 79,928 reads/sample Ribosomal Database Project KEGG	**Mice exposed to restraint stress receiving MT (n** = **5) compared with mice exposed to restraint stress without MT (n** = **5)** • No difference in richness and diversity (number of OTUs, ACE, Chao1, Shannon, and Simpson indices) • Lower relative abundances of *Bacteroides* and *Tyzzerella*, and higher relative abundance of *Mucispirillum, Oscillibacter*, and *Peptococcus* • Higher relative abundance of microbiotas involved in tryptophan metabolism, benzoate degradation and synthesis and degradation of ketone body pathways, and lower relative abundance of microbiotas involved in alanine, aspartate and glutamate metabolism, glutamate metabolism, GABAergic synapses and central carbon metabolism in cancer pathways	**Mice exposed to restraint stress receiving MT compared (n** = **12) with mice exposed to restraint stress without MT (n** = **12)** • Higher corticosterone levels, lower MT levels, and higher reactive oxygens species plasma • Lower expression of TLR2, TLR4, p-p65, and p-IκB in colon
Rong et al.^151^ Mice with jetlag China 2021	8w-old male C57BL/6 mice, standard diet 12 h light/12 h dark cycles (or specific cycles to induce jetlag) 6 jetlag 6 jet lag and MT (0.4 mg/mL) in drinking water 6 MT (0.4 mg/mL) in drinking water 6 controls	Stool nr 16S rRNA gene sequencing V3, V4, nr MiSeq (*Illumina*) nr, nr nr N/A	**Jet-lagged mice receiving MT (n** = **6) compared with jet-lagged mice without MT (n** = **6)** • Lower diversity (Shannon index), no difference in Simpson index • Lower relative abundance of *Enterobacteriales* • Higher relative abundance of *A. muciniphila*	**Jet-lagged mice receiving MT (n** = **6) compared with jet-lagged mice without MT (n** = **6)** • Lower body weight and lipid intake • Lower lipid accumulation in ileum • Lower epididymal white adipose tissue around epididymis • Lower lipopolysaccharides in serum • Lower expression of TLR4, MyD88, and nuclear factor interleuktin-3-regualted protein in ileum • Restored circadian amplitude of nuclear receptor subfamily 1 group D member 1 and nuclear factor interleuktin-3-regualted protein • Lower TLR4/IL-22/STAT3 signalling pathway activity and higher angiopoietin-like 4 levels in ileum
Zhang et al.^150^ Mice exposed to ochratoxin A China 2021	6w-old male C57BL/6 mice, standard diet 12 h light/12 h dark cycles 15 ochratoxin A (250 ug/kg/d) orally for 3w 15 ochratoxin A (250 ug/kg/d) and MT (15 mg/kg/d) orally for 3w 15 controls 15 antibiotics (vancomycin 0.5 g/L, ampicillin 1 g/L, gentamicin 1 g/L, and streptomycin 1 g/L) orally for 3w 15 antibiotics (vancomycin 0.5 g/L, ampicillin 1 g/L, gentamicin 1 g/L, and streptomycin 1 g/L) and ochratoxin A (250 ug/kg/d) orally for 3w 15 antibiotics (vancomycin 0.5 g/L, ampicillin 1 g/L, gentamicin 1 g/L, and streptomycin 1 g/L), ochratoxin A and MT (15 mg/kg/d) (250 ug/kg/d) orally for 3w	Colon content nr 16S rRNA gene sequencing V3, V4, nr MiSeq (*Illumina*) nr, nr nr N/A	**Ochratoxin A-exposed mice receiving MT (n** = **8) compared with ochratoxin A-exposed mice without MT (n** = **8)** (not in antibiotic treated mice) • Higher richness and diversity (ACE, Chao1, Shannon, and Simpson indices) • Higher relative abundance of Bacteroidetes, and lower relative abundance of Bacillota • Lower relative abundance of *Erysipelotrichales*, and higher relative abundances of *Bacteroidales* and *Clostridiales* • Lower relative abundances of *Lactobacillus* and *Desulfovibrio*, and higher relative abundance of *Bacteroides*	**Ochratoxin A-exposed mice receiving MT (n** = **15) compared with ochratoxin A-exposed mice without MT (n** = **15)** (not in antibiotic-treated mice) • Higher weight gain and feed intake • Lower liver to body weight ratio • Lower levels of alkaline phosphatase, gamma-glutamyl transferase, low-density lipoprotein, and triglycerides, and higher levels of total protein, albumin, creatinine kinase in serum • Higer levels of antioxidant enzyme activities and lower levels of maleic dialdehyde in liver • Lower levels of TNF-α, IL-6, IL-1ß, and lipopolysaccharides in liver and ileum • Lower levels of reactive oxygen species, and apoptotic and necrotic cells in liver • Higher mitochondrial membrane potential and adenosine triphosphate in liver • Higher levels of short-chain fatty acids, lower levels of lipopolysaccharides in colon • Restored ileal morphology and permeability (disrupted by ochratoxin A) • Restored protein-expression related to apoptosis, autophagy, antioxidants, inflammation and tight junctions in liver (disrupted by ochratoxin A) • Restored ileum permeability (disrupted by ochratoxin A)
Gao et al.^109^ Mice with sleep deprivation China 2020	Adult male ICR-CD1 mice, standard diet 14 h light/10 h dark cycles 5 sleep-deprived for 3 d 5 sleep-deprived and MT (20 mg/kg/d) intraperitoneally for 3 d 5 sleep-deprived and MT (40 mg/kg/d) intraperitoneally for 3 d 5 controls	Jejunal content QIAamp DNA Stool Mini Kit (*Qiagen)* 16S rRNA gene sequencing V3, V4, composite specific primers MiSeq (*Illumina*) nr, mean 75,221 reads/sample nr KEGG	**Sleep-deprived mice receiving MT (n** = **10) compared with sleep-deprived mice without MT (n** = **5)** • Higher richness and diversity (number of OTUs, ACE, Chao1, and Shannon indices), lower Simpson index • Higher relative abundance of Bacillota, and lower relative abundance of Bacteroidetes • Higher relative abundances of *Bacteroidacea* and *Prevotellaceae*, and lower relative abundances of *Moraxellaceae* and *Aeromonadaceae* • Lower abundance of pathways of cell wall/membrane/envelope biogenesis, energy production and conversion, replication, recombination and repair and translation, ribosomal structure and biogenesis, and higher abundance of pathway of carbohydrate transport and metabolism and transcription	**Sleep-deprived mice receiving MT (n** = **10) compared with sleep-deprived mice without MT (n** = **5)** • Lower cortisol levels in plasma • Higher levels of IL-22, and lower levels of IL-17 and reactive oxygen species in jejunum
Kim et al.^143^ Mice with DSS-induced colitis China 2020	8 to 9w-old male BALB/c and WT/TLR4 KO mice, standard diet 14 h light/10 h dark cycles BALB/c: 10 2.5%-DSS in drinking water for 6 d 10 2.5%-DSS in drinking water plus MT (10 mg/kg/d) intraperitoneally for 8 d 10 MT (10 mg/kg/d) intraperitoneally for 8 d 10 controls TLR KO: 10 2.5%-DSS in drinking water for 6 d 10 2.5%-DSS in drinking water plus MT (10 mg/kg/d) intraperitoneally for 8 d 10 MT (10 mg/kg/d) intraperitoneally for 8 d 10 controls	Stool FastDNA Spin Kit for Soil (*MP Biomedicals*) 16S rRNA gene sequencing V3, V4, nr nr nr, nr EzTaxon-e N/A	**Mice with colitis receiving MT (n** = **5) compared with mice with colitis without MT (n** = **5)** • Higher richness (Chao index) • Lower relative abundance of Pseudomonadota, trend towards higher relative abundance of Bacillota, and trend towards lower relative abundance of Bacteroidetes • Higher relative abundance of *Ruminococcaceae*	**Mice with colitis receiving MT (n** = **10) compared with mice with colitis without MT (n** = **10)** (not in TLR4-knock-out mice) • Less colitis symptoms (lower disease activity index) • Lower histological pathology score in colon • Higher number of goblet cells in colon • Less colon shortening • Higher number of goblet cells in colon • Lower levels of IL-1ß, IL-17α, and higher levels of MT receptors and regenerating islet-derived protein 3-ß in colon • Higher expression of MT receptors in colon
Park et al.^144^ Mice with sleep deprivation exposed to water stress Korea 2020	9w-old male C57BL/6 mice, standard diet 12 h light/12 h dark cycles 6 exposed to water stress for 10 d 6 exposed to water stress and MT (10 mg/kg/d) intraperitoneally for 10 d 6 exposed to water stress and sleep-deprived for 10 d 6 exposed to water stress, sleep-deprived and MT (10 mg/kg/d) intraperitoneally for 10 d 6 controls	Stool samples nr 16S rRNA gene sequencing V3, V4, 341 F, 805 R MiSeq (*Illumina*) EzTaxon-e	**Mice exposed to water stress ± sleep-deprivation receiving MT (n** = **6) compared with mice exposed to water stress ± sleep-deprivation without MT (n** = **12)** • No difference in richness or diversity (number of OTUs, Shannon, and Simpson indices) • Lower relative abundances of *Erysipelotrichaceae* • Higher relative abundances of *Lactobacillus* • Higher relative *A. muciniphila*, and lower relative abundances of *P. massiliensis*	**Mice exposed to water stress ± sleep-deprivation** ± **MT (n** = **24) compared with controls (n** = **6)** • Lower weight, no difference in colon length or inflammation
Gao et al.^108^ Mice with sleep deprivation China 2019	8w-old male CD1 mice, standard diet 14 h light/10 h dark cycles 24 sleep-deprived for 3 d 24 sleep-deprived and MT (20 mg/kg/d) intraperitoneally for 3 d 24 sleep-deprived and MT (40 mg/kg/d) intraperitoneally for 3 d 24 controls	Colon content QIAamp DNA Stool Mini Kit (*Qiagen)* 16S rRNA gene sequencing V3, V4, nr MiSeq (*Illumina*) nr, mean 74,133 reads/sample nr	**Sleep-deprived mice receiving MT (n** = **10) compared with sleep-deprived mice without MT (n** = **5)** • Higher richness (number of OTUs, ACE, Chao1, and Shannon indices), lower Simpson index • Lower relative abundance of Bacillota, and higher relative abundance of Bacteroidetes • Higher relative abundance of *Akkermansia, Bacteroides*, and *Faecalibacterium*, and lower relative abundance of *Aeromonas*	**Sleep-deprived mice receiving MT (n** = **24) compared with sleep-deprived mice without MT (n** = **24)** • Lower norepinephrine levels in plasma • Higher levels of anti-inflammatory (IL-5, IL-10, and IFN-y) and lower levels of pro-inflammatory cytokines (TNF-α, IL-1ß, and IL-6) in plasma • Higher levels of antioxidant enzymes (glutathione peroxidase, superoxide dismutase, and catalase), lower total antioxidant capacity, and lower malondialdehyde levels (lipid peroxidation) in colon • Higher number of goblet cells, proliferation cell nuclear antigen-positive cells, mucin 2 expression, and tight junction protein expression (claudin-1, occluding, zonula occludens-1) in colon
Jing et al.^145^ Mice with spinal cord injury China 2019	Adult female C57BL/6 mice, standard diet 12 h light/12 h dark cycles 16 spinal cord injury 16 spinal cord injury and MT (10 mg/kg twice daily) intraperitoneally for 4w 16 MT (10 mg/kg twice daily) intraperitoneally for 4w 16 controls 6 antibiotics (ampicillin 0.2 g/L, neomycin 0.2 g/L, metronidazole 0.2 g/L, vancomycin 0.1 g/L) 2w before and 4w after spinal cord injury 6 antibiotics (ampicillin 0.2 g/L, neomycin 0.2 g/L, metronidazole 0.2 g/L, vancomycin 0.1 g/L) 2w before and 4w after spinal cord injury and MT 10 mg/kg twice daily intraperitoneal 4w	Stool samples E.Z.N.A. Stool DNA Kit (*Omega Bio-Tek*) 16S rRNA gene sequencing V3, V4, 338 F, 806 R MiSeq (*Illumina*) 2×300, mean 33,999 reads/sample SILVA (SSU128) N/A Culture Columbia blood agar, 38°C, 48 h	**Mice with spinal cord injury receiving MT (n** = **5) compared with mice with spinal cord injury without MT (n** = **5)** • Lower richness and diversity (ACE and Shannon indices) • Lower relative abundance of *Clostridiales*, higher relative abundance of *Lactobacillales (Lactobacillus)* **Mice receiving MT (n** = **5) compared with controls without spinal injury or MT (n** = **5)** • No difference in microbiota composition	**Mice with spinal cord injury receiving MT (n** = **16) compared with mice with spinal cord injury without MT (n** = **16)** • Higer intestinal barrier integrity (tight junctions) and intestinal motility, reduced expression levels of proinflammatory cytokines (IL-17, IFNy, and monocyte chemoattractant protein-1) • Improved weight gain and metabolic profiling • Promoted locomotor recovery **Mice with spinal cord injury without MT (n** = **16) compared with controls without spinal injury or MT (n** = **16)** • Lower levels of MT in colonic tissue • Increased intestinal permeability
Yin et al.^146^ Mice on high-fat diet China 2018	Adult female ICR mice, standard or high-fat diet 12 h light/12 h dark cycles 10 high-fat diet for 2w 10 high-fat diet and MT (0.4 mg/mL) in drinking water ( = approximately 108 mg/kg/d) for 2w 10 controls	Stool samples nr 16S rRNA gene sequencing V3, V4, MiSeq (*Illumina*) nr, mean 72,497 reads/sample SILVA KEGG	**Mice on high-fat diet receiving MT (n** = **8) compared with mice on high-fat diet without MT (n** = **8)** • Higher diversity (Shannon and Simpson indices), no difference in richness (number of OTUs) • Lower relative abundance of Bacillota, and higher relative abundance of Bacteroidetes • Lower relative abundances of *Lactobacillales*, and higher relative abundances of *Bacteroidales, Erysipelotrichales*, and *Burkholderiales* • Lower relative abundances of *Lactobacillus*, *Bacteroides, Alistipes*, and *Parasutterella* • Higher metabolism of amino acid, energy, and cofactors and vitamins, lower membrane transport and carbohydrate metabolism, and lower relative abundance of lipid metabolism-related genes	**Mice on high-fat diet receiving MT (n** = **10) compared with mice on high-fat diet without MT (n** = **10)** • Lower weight of subcutaneous adipose tissue • Lower lipid accumulation in liver • Lower levels of triglycerides in serum • Higher mRNA expression of peroxisome proliferator-activated receptor alpha and liver X receptor beta, and lower mRNA expression of sterol regulatory element-binding protein 2 in subcutaneous adipose tissue • Higher mRNA expression of peroxisome proliferator-activated receptor alpha, liver X receptor beta, and acetyl-coenzyme A carboxylase in liver
Zhu et al.^147^ Mice with DSS-induced colitis China 2018	8w-old ICR mice, standard diet 12 h light/12 h dark cycles 10 5%-DSS in drinking water for 1w 10 5%-DSS and MT (0.2 mg/L) in drinking water for 1w	Colon content nr 16S rRNA gene sequencing V3, V4, 515 F, 907 R MiSeq (*Illumina*) 2×250, mean 8,198 reads/sample SILVA (SSU115) N/A	**Mice with colitis receiving MT (n** = **10) compared with mice with colitis without MT (n** = **10)** • No difference in richness or diversity (ACE, Chao1, Shannon, and Simpson indices) • Higher relative abundance of Bacillota, and lower relative abundance of Bacteroidetes • Higher relative abundances of *Coprococcus*_1 and *Ruminococcaceae*_UCG-014	**Mice with colitis receiving MT (n** = **10) compared with mice with colitis without MT (n** = **10)** • Higher antioxidant capability in plasma (measured by 2,2’-azino-bis3-ethylbenz-thiazoline-6-sulfonic acid radical cation decolorization assay)
Ren et al.^148^ Weanling mice China 2017	3w-old male ICR mice, standard diet 12 h light/12 h dark cycles 10 MT (0.2 g/L) in drinking water for 2w 10 controls 10 antibiotics (streptomycin 1 g/L, ampicillin 1 g/L, gentamicin 1 g/L, and vancomycin 0.5 g/L) orally for 2w 10 antibiotics (streptomycin 1 g/L, ampicillin 1 g/L, gentamicin 1 g/L, and vancomycin 0.5 g/L) and MT (0.2 g/L) orally for 2w 10 germ-free mice 10 germ-free mice receiving MT (0.2 g/L) orally for 2w	Stool samples QIAamp DNA Stool Mini Kit (*Qiagen)* 16S rRNA gene sequencing V3, V4, 338 F, 806 R MiSeq (*Illumina*) 2×250, nr SILVA KEGG	**Mice receiving MT (n** = **8) compared with controls without MT (n** = **8)** • Higher richness (number of OTUs, ACE, and Chao1 indices) • Lower relative abundances of Bacteroidetes • Lower relative abundance of *Bacteroidales* • Lower relative abundance *Prevotellaceae* • Higher relative abundance of *Lactobacillus* (*L. intestinalis, L. johnsonii*, and *L. reuteri)* • Altered microbial metabolism, including amino acid metabolism, polycyclic aromatic hydrocarbon degradation, degradation of aromatic compounds and drug metabolism (4-aminobenzoic acid, 2-indanone, p-cresol, malonamide, saccharic acid, 4-hydroxybenzoic acid, threitol, 7,8-dimethylalloxazine, 6-hydroxy caproic acid dimer, 6-methylmercaptopurine, 5′-methylthioadenosine, palatinitol, cis-2- hydroxycinnamic acid, 2-carboxybenzaldehyde, and saccharopine)	**Mice receiving MT (n** = **10) compared with controls without MT (n** = **10)** • Higher weight gain (not in antibiotic-treated or germ-free mice) • Improved intestinal morphology (villus length, crypt depth, villus to crypt ratio) (not in antibiotic-treated or germ-free mice) • Lower bacterial load in mice infected with enterotoxigenic *E. coli* (not in antibiotic-treated mice)
Xu et al.^149^ Mice on high-fat diet China 2017	10w-old male C57BL/6 J mice, standard diet or high-fat diet 12 h light/12 h dark cycles 5 high-fat diet for 10w 5 high-fat diet plus MT (50 mg/kg/d) orally for 10w 5 controls	Stool samples QIAamp DNA Stool Mini Kit (*Qiagen)* 16S rRNA gene sequencing V3, V4, composite specific bacterial primers MiSeq (*Illumina*) nr, mean 60,983 reads/sample nr N/A	**Mice on high-fat diet receiving MT (n** = **5) compared with mice on high-fat diet without MT (n** = **5) or mice on standard diet without MT (n** = **5)** • Lower richness and diversity (lower number of OTUs, lower ACE, Chao1, and Shannon indices), higher Simpson index • Lower relative abundance of Bacillota, higher relative abundance of Bacteroidetes and higher relative abundance of Verrucomicrobiota • Lower relative abundance of *Desulfovibrionaceae* (only compared with mice without MT on high-fat diet) • Higher relative abundance of *Akkermansia* and lower relative abundaunce of *Alstipes, Anaerotruncus*, and *Helicobacter_marmotae* (only compared with mice without MT on high-fat diet)	**Mice on high-fat diet receiving MT (n** = **5) compared with mice on high-fat diet without MT (n** = **5)** Decreased weight gain, liver steatosis, insulin resistance and low-grade inflammation

ACE – abundance-based coverage estimatorNF-κB – nuclear factor-kappa B

C57BL/6 – C57 Black 6nr – not reported

d – daysMyD88 - myeloid differentiation primary response gene 88

DSS - DSSOTU – operational taxonomic unit

h – hourp-p65 – phosphorylated p65

ICR – institute of cancer researchp-IκB – phosphorylated inhibitor of kappa B

IFN – interferonSTAT3 - signal transducer and activator of transcription 3

IL – interleukinTLR – toll-like receptor

KEGG – Kyoto encyclopedia of genes and genomesTNF-α – tumor necrosis factor alpha

mRNA – messenger ribonucleic nucleic acidw – week

N/A – not applicableWT/TLR4 KO mice – wild-Type/Toll-Like Receptor 4 Knockout mice

Overall, 8 studies (57%) found a higher richness and/or diversity of the intestinal microbiota in mice who were given MT (orally *n* = 5, intraperitoneal *n* = 3)^108,109,139,140,143,146,148,150^, 4 (29%) a lower richness and/or diversity (orally *n* = 2, intraperitoneal *n* = 2)^141,145,149,151^ and 3 (21%) no difference in richness and diversity (orally *n* = 1, intraperitoneal *n* = 1, into colon *n* = 1)^142,144,147^.

**Fig. 2:**
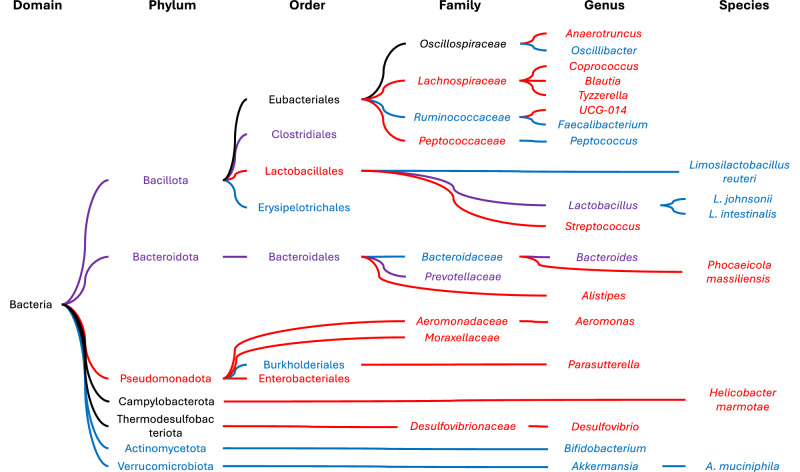
Reported effects of melatonin on the composition of the intestinal microbiota in mice. Blue names/lines indicate a reported increase in abundance; red indicates a decrease; purple indicates both. Black names/lines are shown for clarity and have no reported effects in the literature. Note that all names are adjusted to reflect current taxonomic nomenclature at the time of writing and may not match how these are reported in older literature.

On the phylum level, 5 studies reported a lower relative abundance of Bacillota^108,139,146,149,150^ and higher relative abundance of Bacteroidota^108,139,146,149,150^ in mice who received MT^139^. In contrast, 3 studies each reported higher relative abundances of Bacillota^109,140,147^ and a lower relative abundances of Bacteroidetes^109,147,148^. Furthermore, 2 studies reported higher relative abundances of Verrucomicrobiota^141,149^, and one each a higher relative abundance of Actinomycetota^141^ and a lower relative abundance of Pseudomonadota^143^. On the order level, MT was associated with lower relative abundances of *Enterobacteriales*^150,151^ and *Lactobacillales*^139,145,146^, and higher relative abundances of *Burkholderiales*^108^ and *Erysipelotrichales*^108^. Inconsistent findings were reported for *Clostridiales* (lower^139,145^ and higher^150^) and *Bacteroidales* (higher^108,150^ and lower^148^). On the family level, MT was associated with level lower relative abundances of *Aeromonadaceae*^109^*, Desulfovibrionaceae*^149^*, Erysipelotrichaceae*^144^*, Lachnospiraceae*^141^*, Moraxellaceae*^109^, and *Peptococcaceae*^141^, and higher relative abundances of *Bacteroidaceae*^109^ and *Ruminococcaceae*^143^. Inconsistent findings were reported for *Prevotellaceae*^109,148^. On the genus level, MT was associated with lower relative abundances of *Aeromonas*^108^*, Alistipes*^146,149^, *Anaerotruncus*^149^*, Clostridium*_XIVa^139^, *Coprococcus*_1^147^, *Desulfovibrio*^139,141,150^*, Helicobacter_marmotae*^149^*, Parasutterella*^108^*, Ruminococcaceae*_UCG-014^147^, and *Tyzzerella*^142^, and higher relative abundances of *Akkermansia*^108,149^*, Bifidobacterium*^141^*, Blautia*^140^*, Faecalibacterium*^108^*, Mucispirillum*^142^*, Oscillibacter*^142^*, Peptococcus*^142^, and *Streptococcus*^142^. Inconsistent findings were reported for *Bacteroides*^108,140,142,146,150^ and *Lactobacillus*^139,140,144–146,148,150^. On the species level, MT was associated with a higher relative abundance of *A. muciniphila*^144,151^*, L. intestinalis*^147^*, L. johnsonii*^147^, and *L. reuteri*^147^ and lower relative abundance of *Phocaeicola massiliensis* (Fig. 2)^144^. Interestingly, antibiotics often suppressed the effects of exogenous MT on the intestinal microbiota^150,151^.

MT has also been reported to alter metabolic pathways of the intestinal microbiota. For example, enhancement in pathways related to tryptophan metabolism, benzoate degradation, and synthesis/degradation of ketone bodies and impairment of pathways associated with alanine, aspartate, and glutamate metabolism, as well as pathways related to GABAergic synapses and central carbon metabolism in cancer pathways^142^. Furthermore, impairment of pathways involved in cell wall/membrane/envelope biogenesis, energy production, recombination, repair along with translation, and ribosomal structure biogenesis have been observed after MT supplementation^143,148^. Furthermore, enhancement of pathways involved in amino acids and cofactors/vitamins have been reported, while pathways involved in carbohydrate metabolism findings were inconsistent^146,148^.

## Effects of intestinal MT on the intestine, other organs, and diseases

MT has multiple effects on the intestine through interactions with MT, serotonin, and cholecystokinin B receptors^152^, as well as receptor-independent processes. It inhibits the increase in gastrointestinal motility and smooth muscle cell contraction caused by serotonin^153^. In humans, MT has been shown to influence intestinal permeability^47^, while in rats, it has been reported to influence intestinal motility^154–157^, permeability^155,156^, bicarbonate secretion^155,156^, and the release of peptides involved in energy balance^158^, similar to observations in sheep^159^.

In mice and rats with induced ulcerative colitis, MT decreases inflammation-associated mucosal injury by inhibiting NF-kB activation, regulating macrophages activity, and reducing chemokine expression, bacterial translocation, and apoptosis^160–164^. MT also protects rats from radiotherapy-induced small intestine toxicity^165^. In humans with ulcerative colitis or proctitis, increased numbers of enterochromaffin cells, elevated ASMT expression, and higher levels of 6-sulfatoxy MT in urine indicate enhanced MT secretion^166,167^. Similarly, increased MT secretion has been observed in patients with lymphocytic colitis^168^.

There is only limited and indirect evidence from animal studies regarding how MT produced in the intestine affects other organs. One study in mice suggests that MT treatment improves cognitive function and intestinal histological findings following cerebral damage induced by intestinal ischemia and reperfusion^169^. Similarly, a study in rats showed that MT mitigates oxidative and nitrosative stress in both the intestine and the lung in response to mesenteric ischemia and reperfusion injury^170^. Another study in rats showed that MT exerts protective effects on the stomach and pancreas, preventing damage and promoting healing of gastric ulcers through antioxidant mechanisms and the activation of specific receptors^171^.

## Discussion

MT and the intestinal microbiota have a complex functional interrelationship. Intestinal MT is derived from various potential sources, including food, enterochromaffin cells, and the microbiota itself (Fig. 1); however, the contributions from these sources to overall intestinal MT levels require further investigation. Once produced, MT has been shown to directly influence the growth and metabolism of intestinal microbes, affecting community diversity and abundance at various taxonomic levels. Interestingly, MT also induces intestinal microbiota remodeling, improving intestinal health by reducing oxidative stress, autophagy, and inflammation—a phenotype transferable via fecal microbiota transplantation^172^. The interaction between MT and microbes appears to be mediated indirectly through host immune responses and microbial modulation. Further research is needed to clarify whether microbes possess specific receptors for MT. The impact of MT, particularly intestinal MT, on health outcomes remains poorly characterized, and its interaction with beneficial and pathogenic microbes requires further elucidation.

MT is widely used as treatment for different populations and diagnoses, including insomnia, sleep regulation issues in individuals with blindness or traumatic brain injury, and neurological or psychiatric disorders^173–176^. In sepsis, characterized by inflammation, oxidative stress, and mitochondrial dysfunction, MT has shown beneficial effects, significantly reducing inflammatory biomarkers and improving sepsis outcomes in neonates^177,178^, and in mice infected with *S. aureus* and *Escherichia coli*, MT administration resulted in better clearance of the bacteria from blood^179^. An in vitro study suggested that MT’s efficacy in sepsis may be due to its ability to reduce mitochondrial dysfunction, oxidative stress, and cytokine responses^180^.

In humans, evidence regarding the effect of MT on activity of inflammatory bowel disease is conflicting. Some case reports show symptom improvement with MT intake^181^, while others report aggravation^182^. Patients with ulcerative colitis have lower colonic MT levels, which negatively correlated with disease severity^141^. A small cohort study found improved biopsy results in patients with inflammatory bowel disease who received MT alongside regular treatment^183^. In children with neurodevelopmental abnormalities, MT improved gastrointestinal symptoms, including gastro-esophageal reflux, ulcerative colitis, and non-specific chronic diarrhea^184^. Probiotics may influence MT production, as symptom improvement in patients with irritable bowel syndrome correlates with higher morning MT levels following probiotic intake^185^.

In animal models, MT has been shown to prevent obesity^186–190^. A decreased abundance of Bacteroidetes and increased abundance of Bacillota has been found in obese rodents^191,192^ and humans^193^. In mice fed a high-fat diet, MT supplementation significantly increased Bacteroidetes and decreased Bacillota levels^149^. MT supplementation was also associated with an increased abundance of *Akkermansia*, a bacterial genus that has been associated with improved metabolic health, such as decreased rates of obesity, diabetes and inflammation in rodents^194–196^ and humans^197,198^. Similarly, MT supplementation was associated with lower abundances of *Alistipes and Anaerotruncus*, both of which have been associated with obesity^199,200^.

Shift work is associated with systemic inflammation and a higher risk for cancers, allergies and autoimmune diseases^201,202^. Shift workers have a higher incidence of breast and prostate cancers, potentially linked to chronically reduced MT levels^203–205^. Night shift workers also show increased markers of systemic inflammation, including increased leukocyte counts^206^, and are at higher risk for high blood pressure^207^ and metabolic syndrome^206–209^. Sleep deprivation is linked to a higher risk for ulcerative colitis^202^, possibility due to MT suppression^108^. MT attenuates intestinal microbiota dysbiosis (such as reduced diversity, lower abundances of *Akkermansia, Bacteroides*, and *Faecalibacterium*, and a higher abundance of *Aeromonas)* induced by sleep deprivation in mice^108,109^, while improving the intestinal barrier function^108,140,145^. Furthermore, MT reverses sleep deprivation-induced intestinal microbiota dysbiosis by suppressing oxidative stress and inflammation^109^.

MT might be a valuable therapeutic agent for dysbiosis-related conditions and disturbances in the gut-brain axis, affecting both gastrointestinal health and neurological functions. Current studies focus on engineering *E. coli*^210–212^ and *S. cerevisiae*^213^ to synthesize large amounts of MT. However, more research is required to identify which intestinal microbes produce MT, their interactions with other microbes, and whether co-administration of MT with synbiotics could enhance health outcomes. It remains unclear whether MT-producing microbes self-regulate their functions or if environmental MT influences the cellular regulation of various microbial species. This distinction is significant as it could impact our understanding of microbial behavior and their interactions with the host environment.

## Data Availability

No datasets were generated or analyzed during the current study.
